# A Survey of Upper Extremity Musculoskeletal Ultrasound Use in a Surgical Practice

**DOI:** 10.1177/22925503241285459

**Published:** 2024-10-03

**Authors:** Mieke Heyns, Maleka Ramji, Aaron Knox, Justin Yeung

**Affiliations:** 1 Section of Plastic Surgery, Chinook Regional Hospital, Calgary, Canada; 2 Section of Plastic Surgery, 2129 University of Calgary, Calgary, Canada

**Keywords:** ultrasonography, upper extremity, hand, wrist, peripheral nerves, échographie, membres supérieurs, mains, poignet, nerfs périphériques

## Abstract

**Purpose:** The purpose of this study is to gather the practices and perceptions among upper extremity surgeons regarding the use of musculoskeletal ultrasound for diagnostic and therapeutic intervention. **Methods:** A 36-question survey was developed from a literature review and author consensus. This survey was then piloted among a small group of hand surgeons prior to distribution. The survey included respondent characteristics, use of ultrasound in their current practice, referral patterns for ultrasound, and interest in incorporating ultrasound into practice and residency training. The refined survey was distributed to the Canadian Society of Plastic Surgeons, and Wrist and Elbow Society of Canada, as well as plastic surgery training programs. A reminder email was sent at 3 weeks and again at 8 weeks. **Results:** There were 152 responses after 505 survey invitations (30% response rate). Of these responses, 140 were complete (92%). Diagnostic ultrasound was used by 16 respondents (11%) for a myriad of pathologies. Only 5% used ultrasound for guided injections in a clinic or office setting. Money, time, lack of training, and lack of billing code were the major barriers to ultrasound implementation for 124 (89%) hand surgeons. Most respondents (84%) believe that ultrasound training should be incorporated into residency training. Ninety-one respondents (60%) are interested or very interested in incorporating ultrasound into their current practice. **Conclusions:** Ultrasound is a valuable resource that is seldom used as a point of care by Canadian hand surgeons due to several barriers. Survey results suggest that upper extremity surgeons are keen to have ultrasonography be part of residency education and most wish to adopt it into their future practice.

## Résumé

**Introduction :** La présente étude vise à connaître les pratiques et les perceptions des chirurgiens des membres supérieurs au sujet de l’utilisation de l’échographie musculosquelettique lors d’interventions diagnostiques et thérapeutiques. **Méthodologie :** Un sondage de 36 questions a été créé à partir d’une analyse bibliographique et du consensus des auteurs. Ce sondage a ensuite fait l’objet d’un projet pilote auprès d’un petit groupe de chirurgiens de la main avant d’être distribué. Il contenait des questions sur les caractéristiques des répondants, le recours à l’échographie dans leur pratique, leurs modes de consultation en échographie ainsi que leur intérêt à intégrer l’échographie à leur pratique et à la formation en résidence. Le sondage amélioré a été distribué aux membres de la Société canadienne des chirurgiens plasticiens et de la Société du poignet et du coude du Canada, de même qu’aux programmes de formation en chirurgie plastique. Ces groupes ont reçu un courriel de rappel au bout de trois semaines, puis de huit semaines. **Résultats :** Les auteurs ont reçu 152 réponses sur 505 invitations au sondage (taux de réponse de 30 %). De ce nombre, 140 étaient complets (92 %). Ainsi, 16 répondants (11 %) utilisaient l’échographie diagnostique, pour une myriade de pathologies. Seulement 5 % y recouraient pour les injections guidées en clinique ou en cabinet. Pour 124 des chirurgiens de la main (89 %), les coûts, le temps, le manque de formation et l’absence de code de facturation représentaient des obstacles majeurs à l’adoption de l’échographie. La plupart des répondants (84 %) étaient d’avis que la formation en échographie devrait être intégrée à la formation en résidence. Enfin, 91 répondants (60 %) étaient intéressés ou très intéressés à intégrer l’échographie à leur pratique. **Conclusion :** L’échographie est une ressource précieuse que les chirurgiens de la main utilisent peu au point de service en raison de plusieurs obstacles. D’après les résultats du sondage, les chirurgiens des membres supérieurs aimeraient que l’échographie fasse partie de la formation en résidence, et la plupart souhaitent l’adopter dans leur future pratique.

## Introduction

Musculoskeletal (MSK) ultrasound (US) is a rapidly evolving technique that is gaining popularity for the evaluation and treatment of a myriad of upper extremity pathologies.^1^ Its applications include assessment of soft tissue tumors, tendon and ligament pathology, as well as nerve compression syndromes. Dynamic assessment can evaluate tendon glide or the presence of nerve subluxation.^2–4^ There are also therapeutic applications that involve the treatment of compression neuropathies with guided corticosteroid injections for symptom relief that show better results than landmark or palpation-guided injection strategies.^5–10^ The accuracy of MSK US is operator-dependent; hence its clinical use has traditionally been limited to non-surgical specialties such as radiology and physiatry.^11^

Recently, there has been expanding accessibility and affordability of portable high-resolution MSK US devices. In this light, upper extremity surgeons can harness this increasing accessibility to technology and implement MSK US throughout the broad scope of practice.^12^ Furthermore, MSK US of the upper extremity depicts subtle structural changes requiring a nuanced understanding of relevant anatomy and its surgical application.^13^ It is undeniable that upper extremity surgeons are best equipped to perform and interpret MSK US images due to their intimate familiarity with this anatomy and biomechanical function.^14–16^ Given the utility of this imaging modality and the current lack of adoption by hand surgeons in Canada, it is surprising that its perception and barriers to implementation have not been formally surveyed among Canadian upper extremity surgeons. The purpose of this study is to gather the current practices and perceptions among upper extremity surgeons regarding the use of MSK US for diagnostic and therapeutic intervention. We are also curious about the reasons for not using this imaging modality and the barriers to implementing ultrasonography as a clinical tool.

## Methods

After obtaining ethics approval from the University of Calgary IRISS review board, a 36-question survey was created using SurveyMonkey™ survey software. The survey consisted of questions to assess respondent demographic characteristics, US practices, and barriers to implementation. The questions were divided into 4 subgroups:

1. Information on current practice type, duration, and location. Demographics regarding the level of training, fellowship completion, and type. Prevalence of formal US training.
2. Use of US in current practice, setting, and frequency; type of diagnostic and therapeutic uses. Those who answered “no” to using US in their practice were asked questions regarding barriers to implementation.
3. Reasons for referral for US assessment of patients and for which pathologies patients were being referred. Again, barriers to use or reasons around not using US were sought in this question stem.
4. Respondents were surveyed about their interest in incorporating US into daily surgical practice and surgical residency training. Ideas around how to best teach and introduce US into the education curriculum were sought from respondents.

After the completion of the survey, the survey was distributed by email invitation to practicing upper extremity surgeons in Canada in May 2021. It was sent to all registered members of the Canadian Society of Plastic Surgeons (CSPS) and the largely orthopedic hand-represented group, the Wrist and Elbow Society of Canada (WECAN). The survey was also sent to each of the residency programs for dissemination to their residents and fellows. Participation was voluntary and access to the survey was granted by a link in the email. A reminder email was sent at 3 weeks and another at 8 weeks.

## Results

A total of 505 email invitations were sent to members of the CSPS, WECAN, and plastic surgery trainees across Canada. We received 152 responses, yielding a response rate of 30%. Of these responses, 140 were complete, giving a completion rate of 92%. Only complete responses were used in data collection and analyses.

### Respondent Demographics

One hundred and sixteen participants (83%) had a background in Plastic Surgery, versus only 24 (17%) with Orthopedic Surgery training (Table 1). Most of the respondents were staff surgeons (108, 77%), compared to 32 (23%) who were residents or fellows. Sixty-five (46%) participants excluding trainees had either completed or are currently participating in a hand and upper extremity fellowship. Seventeen respondents had not gone on to complete any fellowship-level subspecialization.

**Table 1.** Respondent's Residency and Fellowship Background.

	*n* (%)
**Residency type**	
Plastic surgery	116 (83)
Orthopedic surgery	24 (17)
** Level of training**	
Resident	25 (18)
Fellow	7 (5)
Staff surgeon	108 (77)
** Fellowship specialty**	
Hand/upper extremity	65 (46)
Other(*)	26 (19)
Breast/microsurgery	14
Craniofacial	9
Medical education	2
Pediatrics	5
No fellowship	17(12)

*Some respondents had completed more than 1 fellowship.

Fifty-two staff surgeons worked in an academic practice, 31 had a community practice, and 24 were identified as having a mixed practice. A single respondent worked exclusively in private practice (Table 2). The majority of staff surgeon respondents have been in practice for more than 5 years (88%), while 29% had been in practice for 11–20 years. Sixty-five percent of respondents had practices based out of Ontario or Alberta (Table 3). British Columbia, Quebec, and the Maritimes had the next most respondents, respectively, with 23 (16%), 12 (9%), and 10 (7%) individuals.

**Table 2.** Staff Surgeon Practice Characteristics.

	n = 108 (%)
**Type of practice**	
Community practice	31 (29)
Academic practice	52 (48)
Mixed practice	24 (22)
Private practice	1 (0.9)
** Years in practice**	
0 to 5	13(12)
6 to 10	21(19)
11 to 20	31(29)
21–30	28(26)
More than 30	14(13)

**Table 3.** Region of Current Practice or Training.

	n = 140 (%)
British Columbia	23 (16)
Alberta	34 (24)
Saskatchewan	2 (1)
Manitoba	1 (0.7)
Ontario	58 (41)
Quebec	12 (9)
Maritimes	10 (7)
Territories (Yukon, NWT, Nunavut)	0 (0)

### Use of the US

Only 11% of the 140 respondents identified as presently using the US in the office or clinic setting. Half of these users were using the US at least once a month, while the other half were using it more frequently, at least once a week or daily. The most common pathologies assessed in the clinic with the US from these respondents, were mass lesion (*n* = 10), tendon (*n* = 9), carpal tunnel syndrome (*n* = 7), evaluating soft tissue infection (*n* = 7), and joint pathology (*n* = 4). With regard to therapeutic interventions, from these respondents, the following interventions were identified: injection of carpal tunnel (*n* = 5), nerve blocks (*n* = 3), first extensor compartment injection (*n* = 3), carpometacarpal arthritis injection (*n* = 2), trigger finger injection (*n* = 1), and other small joint arthritis injection (*n* = 1).

### Referral for US

One hundred and fifteen (82%) surgeons refer patients for diagnostic MSK US services. Common reasons for referring patients include tendon pathology (*n* = 91), joint pathology (*n* = 45), mass lesion (*n* = 36), carpal tunnel syndrome (*n* = 17), ligamentous injury (ie, ulnar collateral ligament) (*n* = 17), cubital tunnel syndrome (*n* = 16), and other which included nerve pathology, infection, bony pathology, and competence of palmar arch and foreign body (*n* = 5).

Forty-six respondents (33%) stated that they refer patients specifically for US-guided injections for treatment of tendonitis (*n* = 23), nerve compression (*n* = 12), arthritis (*n* = 4), ganglion aspiration (*n* = 2), and other (*n* = 1). Reasons listed for not referring patients for ultra-sound guided injections include access to fluoroscopy in the clinic or comfortability with landmark-based methods for injection over image guidance.

### US Training Questions

**Figure 1.**
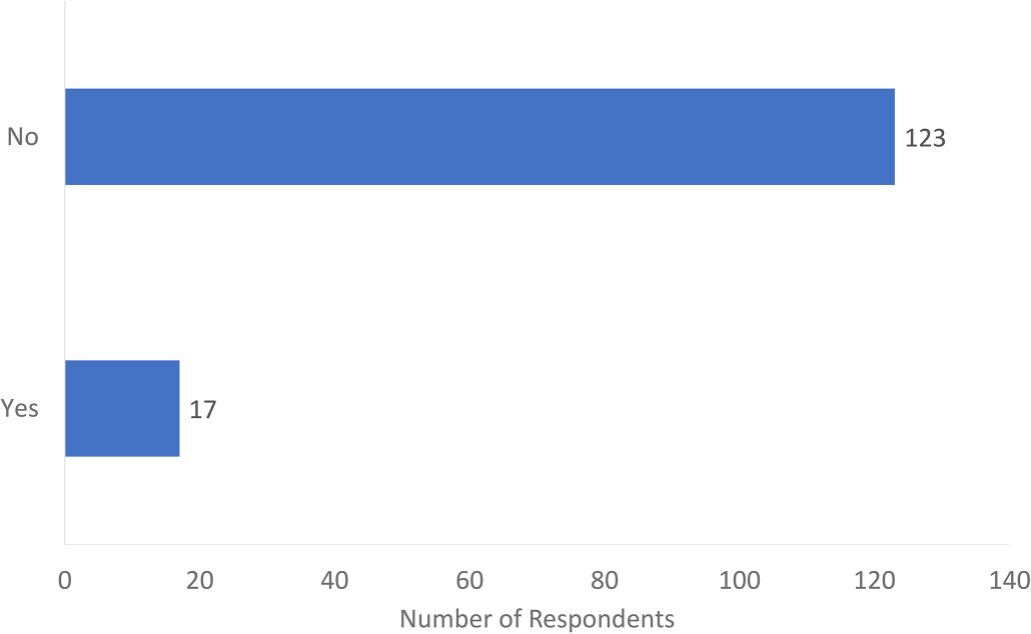
Have you had formal musculoskeletal ultrasound training?

Of the 140 respondents, the majority (123, 88%) had no formal US training, while 17 (12%) did have US training (Figure 1). Of these 17, 13 used an open box to comment on the nature of their US exposure and training which included: attending a US course affiliated with a conference (*n* = 4), completing independent US courses (*n* = 4), attending skills labs at their academic institution (*n* = 3), training during fellowship (*n* = 2), and a single individual had partially completed a radiology residency before transferring to plastic surgery.

**Figure 2.**
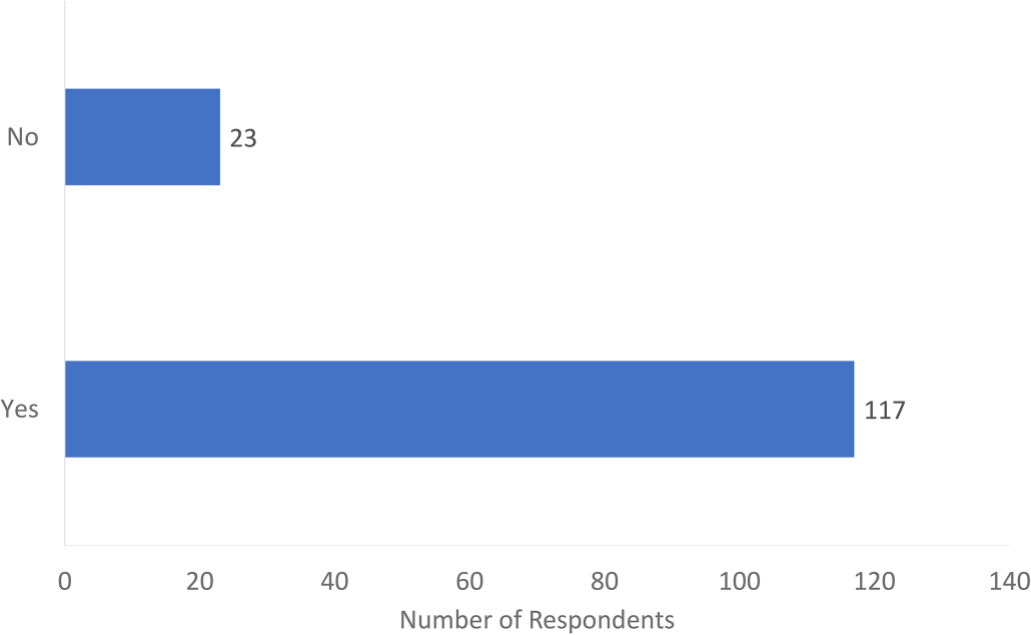
Do you think musculoskeletal (MSK) ultrasound teaching should be provided in surgical residency?

**Figure 3.**
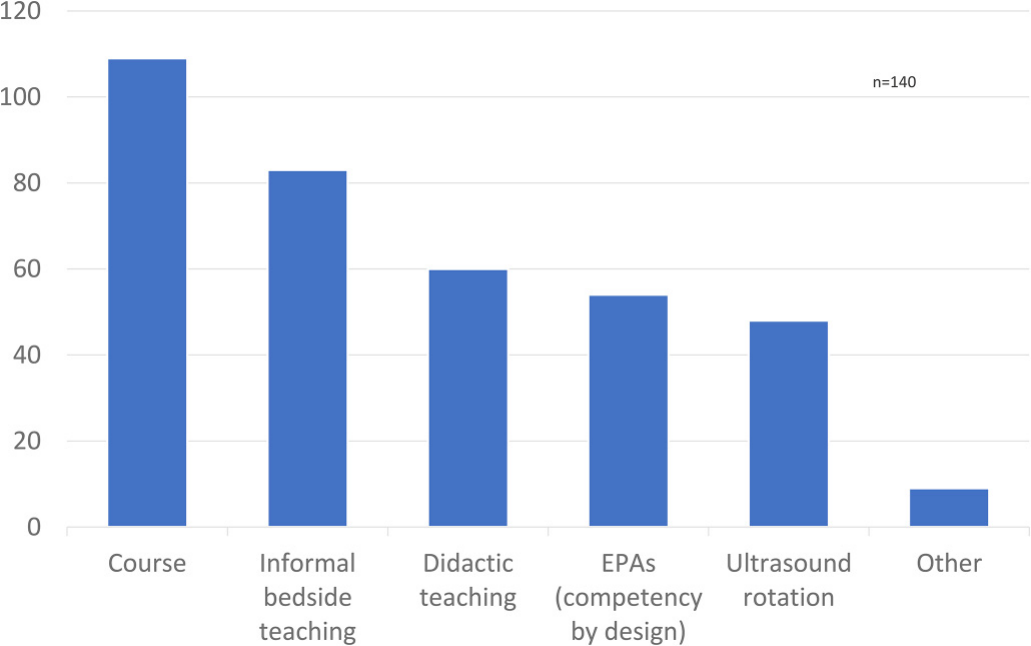
How do you think musculoskeletal (MSK) ultrasound teaching should be incorporated into residency?

Of the 140 respondents, 117 (84%) agreed that MSK US training should be provided as part of the surgical residency educational curriculum (Figure 2). Sixty percent of respondents were interested or very interested in incorporating the US into their practice. Most respondents (*n* = 109) advocated that a course would be the best method for teaching the US to surgical residents (Figure 3). This was followed by informal clinical teaching (*n* = 83), didactic teaching (*n* = 60), incorporation of Entrustable Professional Activities (EPA) (n = 54), and a US-specific rotation (*n* = 48). Of note, respondents were permitted to check multiple answers. Respondents also suggested other teaching methods such as an academic day dedicated to US exposure or collaborating with radiology colleagues to organize an appropriate curriculum.

### Barriers to US use

Of the respondents, the majority (89%) do not use the US in their practice currently. These individuals reported several barriers to US implementation including “I don’t have an ultrasound machine” (*n* = 98), “I don’t have the training to use ultrasound” (*n* = 70), “I only need to ultrasound patients infrequently” (*n* = 33), “I am too busy to use ultrasound” (*n* = 32), “There is no billing code for ultrasound use” (*n* = 32), liability issues (*n* = 16), “I don’t think ultrasound is a valuable modality” (*n* = 5), Price of a US machine (*n* = 3), Others cited that they were not familiar with the breadth of MSK US applications for hand surgery, and would defer to more experienced colleagues in radiology to complete US examination (*n* = 13).

## Interpretation

Although the US is a valuable clinical tool for its accuracy, versatility, and cost-effectiveness, the vast majority of upper extremity surgeons in Canada do not use this imaging modality in a clinical or office setting. Our study confirms that surgeons use the US for both diagnostic and therapeutic interventions but are not doing so themselves and referring to other practitioners. This study also helped identify multiple barriers to the adoption use of US machines by surgeons in the office or clinical setting.

The most cited reason for not personally using a US machine was lack of access to a machine and lack of training in ultrasonography. Other cited reasons included not having a billing code for this intervention or being too busy to perform the US at the time of patient assessment. Understandably, the investment needed to obtain a high-frequency portable US for in-clinic or office use may not be justified for many upper extremity surgeons without a reliable mechanism of reimbursement. To address this, it is worthwhile for hand surgeons to undergo specialized training in the US, ensuring their competence in wielding this valuable imaging tool. Once they have acquired this proficiency, it becomes more justifiable to advocate for including US applications in the fee schedule for hand surgeons. This strategic approach not only ensures that hand surgeons can fully harness the benefits of the US in their practice but also promotes the delivery of more accurate and effective care to patients with hand-related pathologies.^16,17^ With regard to access to a machine, there are many options on the market to consider. In recent years, US manufacturers have been producing high-quality, handheld machines that are cheaper than larger format alternatives. These machines can cost between $5000 to $10,000 and are a manageable investment for the hand surgeon hoping to incorporate the tool more easily into their practice.^18^ Respondents to the survey were overwhelmingly supportive of incorporating US teaching into residency training, and most respondents were keen to incorporate ultrasonography into their own practice as well. At present, our institution has developed 2 courses for the US of the nerve and the tendon at the level of the hand and wrist. The course has been piloted and validated with residents and staff surgeons with the intent to incorporate the course into the plastic surgery residency training curriculum. There are plans to involve relevant stakeholders in this decision including the Royal College Training Program Committees and Program Directors across the country.^19^

There were several limitations to the study, including response bias which is reflected in our survey response rate of 30%. However, because our survey invites were sent mostly to a professional society, participation was voluntary, so a lower survey response rate is not completely unexpected. Our sample size is small and potentially there is a self-selection bias when choosing to complete this survey or not.

## Conclusion

This survey study demonstrates that MSK point-of-care US is not currently performed by many Canadian upper extremity surgeons. The interest in incorporating the imaging tool into clinical practice and point-of-care assessments is noted among the majority of respondents. There are several barriers, however, to its implementation including financial burden and a learning curve to proficiency. To mitigate this, upper extremity surgeons are keen to have US be part of residency education and most wish to adopt it into their future clinical practice.

## References

[1] Bollard SM Kelly B McDermott C Potter S . The use of point of care ultrasound in hand surgery. J Hand Surg Am. 2021;46(7):602-607. 33832787 10.1016/j.jhsa.2021.02.004

[2] Chen SH-T Chen Y-L Cheng M-H Yeow K-M Chen H-C Wei F-C . The use of ultrasonography in preoperative localization of digital glomus tumors. Plast Reconstr Surg. 2003;112(1):115-119. 12832884 10.1097/01.PRS.0000066163.81401.05

[3] Hobson-Webb LD Padua L . Ultrasound of focal neuropathies. J Clin Neurophysiol. 2016;33(2):94-102. 27035249 10.1097/WNP.0000000000000233

[4] Soni P Stern CA Foreman KB Rockwell WB. Advances in extensor tendon diagnosis and therapy. Plast Reconstr Surg. 2009;123(2):52e-57e. 10.1097/01.prs.0000345599.95343.2a 19182570

[5] Hoefler AH Miller EM Kobayashi Y Gottschalk AW . Ultrasound: A useful tool in the diagnosis and localization of ulnar neuropathy at the elbow. Ochsner J. 2021;21(1):3. 33828418 10.31486/toj.21.0002 PMC7993428

[6] Chen P-C Chuang C-H Tu Y-K Bai C-H Chen C-F Liaw M-Y . A Bayesian network meta-analysis: Comparing the clinical effectiveness of local corticosteroid injections using different treatment strategies for carpal tunnel syndrome. BMC Musculoskelet Disord. 2015;16:1-16. 26585378 10.1186/s12891-015-0815-8 PMC4653918

[7] Altinok T Baysal O Karakas H , et al. Ultrasonographic assessment of mild and moderate idiopathic carpal tunnel syndrome. Clin Radiol. 2004;59(10):916-925. 15451352 10.1016/j.crad.2004.03.019

[8] Ooi CC Wong SK Tan AB , et al. Diagnostic criteria of carpal tunnel syndrome using high-resolution ultrasonography: Correlation with nerve conduction studies. Skeletal Radiol. 2014;43:1387-1394. 24915739 10.1007/s00256-014-1929-z

[9] Beekman R Schoemaker M Van Der Plas J , et al. Diagnostic value of high-resolution sonography in ulnar neuropathy at the elbow. Neurology. 2004;62(5):767-773. 15007128 10.1212/01.wnl.0000113733.62689.0d

[10] Kele H . Ultrasonography of the peripheral nervous system. Perspect Med. 2012;1(1-12):417-421.

[11] European Society of Radiology (ESR). Position statement and best practice recommendations on the imaging use of ultrasound from the European Society of Radiology ultrasound subcommittee. Insights Imag. 2020;11(1):115. 10.1186/s13244-020-00919-x PMC7652945 33165666

[12] Díaz HF Fernández FD Horcajadas ÁB Martínez MV Yubero ME. Usefulness of the ultrasound in hand surgery: Part I. Rev Iberoam Cir Mano. 2021;49(2):e128-e139.

[13] Vögelin E. Ultrasonography: The third eye of hand surgeons. J Hand Surg Eur Vol. 2020;45(3): 219-225. 31955646 10.1177/1753193419898155

[14] Vreju FA Ciurea ME Popa D , et al. Ultrasonography in the diagnosis and management of non inflammatory conditions of the hand and wrist. Med Ultrason. 2016;18(1):90-95. 26962560 10.11152/mu.2013.2066.181.vej

[15] Jones M England S Muwanga C Hildreth T . The use of ultrasound in the diagnosis of injuries of the ulnar collateral ligament of the thumb. J Hand Surg: Br Eur Vol. 2000;25(1):29-32. 10.1054/jhsb.1999.0283 10763719

[16] Dickson R Duncanson K Shepherd S . The path to ultrasound proficiency: A systematic review of ultrasound education and training programmes for junior medical practitioners. Australas J Ultrasound Med. 2017;20(1):5-17. 34760465 10.1002/ajum.12039 PMC8409858

[17] Vigny S Rubinstenn E Michelin P , et al. Ultrasound identification of hand and wrist anatomical structures by hand surgeons new to ultrasonographic techniques. Surg Radiol Anat. 2024;46(6):795-804. 38597950 10.1007/s00276-024-03355-4

[18] Le MP Voigt L Nathanson R , et al. Comparison of four handheld point-of-care ultrasound devices by expert users. Ultrasound J. 2022;14(1):27. 35796842 10.1186/s13089-022-00274-6 PMC9263020

[19] Karpinski J Stewart J Oswald A Dalseg TR Atkinson A Frank JR. Competency-based medical education at scale: A road map for transforming national systems of postgraduate medical education. Perspect Med Educ. 2024;13(1):24. 38371306 10.5334/pme.957 PMC10870941

